# Characterization of the *Bacillus cereus* Group Isolated from Ready-to-Eat Foods in Poland by Whole-Genome Sequencing

**DOI:** 10.3390/foods13203266

**Published:** 2024-10-14

**Authors:** Joanna Kowalska, Elżbieta Maćkiw, Dorota Korsak, Jacek Postupolski

**Affiliations:** National Institute of Public Health NIH-National Research Institute, Department of Food Safety, Laboratory of Food Microbiology, 00-791 Warsaw, Poland; emackiw@pzh.gov.pl (E.M.); dkorsak@pzh.gov.pl (D.K.); jpostupolski@pzh.gov.pl (J.P.)

**Keywords:** confectionery, RTE, WGS, toxin profiles, antimicrobial resistance

## Abstract

*Bacillus cereus sensu lato* can contaminate food and cause food poisoning by producing toxins such as cereulide, toxin BL, and cytotoxin K. In this study, we retrospectively analyzed *B. cereus sensu lato* from retail food products and food poisoning cases using PCR methods to determine their virulence profiles. A new toxin profile, encoding all four toxins (*hbl*, *nhe*, *cytK*, *ces*), was found in 0.4% of isolates. The toxin profiles, classified into A-J, revealed that 91.8% harbored *nhe* genes, while *hbl*, *cytK*, and *ces* were detected in 43.8%, 46.9%, and 4.2% of isolates, respectively. Whole-genome sequencing (WGS) identified four distinct species within the *B. cereus* group, with 21 isolates closely related to *B. cereus sensu stricte*, 25 to *B. mosaicus*, 2 to *B. toyonensis*, and 1 to *B. mycoides*. Three novel sequence types (STs 3297, 3298, 3299) were discovered. Antibiotic resistance genes were common, with 100% of isolates carrying beta-lactam resistance genes. Fosfomycin (80%), vancomycin (8%), streptothricin (6%), tetracycline (4%), and macrolide resistance (2%) genes were also detected. These results highlight the genetic diversity and antibiotic resistance potential of *B. cereus sensu lato* strains in Polish food products.

## 1. Introduction

The *Bacillus cereus* group, also known as *B. cereus sensu lato* (*B. cereus s. l.*; *B. cereus* group), is a complex of closely related Gram-positive, aerobic or facultatively anaerobic, spore-forming microorganisms widespread in the environment [1]. The classification of *B. cereus s. l.* isolates into species-level taxonomic units is essential for the evaluation of public health risks and industrial utility. However, the taxonomy of *B. cereus s. l.* has undergone significant revisions in recent decades, resulting in a landscape marked by ambiguity, lack of standardization, and nomenclatural inconsistencies. This complexity poses a considerable challenge to effective communication within the scientific community studying *B. cereus s. l.* [2].

By 2016, *B. cereus s.l.* included nine published species: *B. anthracis, B. cereus sensu stricto* (*B. cereus s. s.*), *B. cytotoxicus*, *B. mycoides*, *B. pseudomycoides*, *B. thuringiensis*, *B. toyonensis*, *B. weihenstephanensis*, and *B. wiedmannii.* Recently, a nomenclature framework combining genomospecies, subspecies, and biovar classifications was proposed by Carroll et al. [2]. According to this, the *B. cereus* group includes twelve genomospecies: *B. bingmayongensis*, *B. cereus s. s.*, *B. clarus*, *B. cytotoxicus*, *B. gaemokensis*, *B. luti*, *B. manliponensis*, *B. mosaicus*, *B. mycoides*, *B. paramycoides*, *B. pseudomycoides*, and *B. toyonensis.*

Some strains within *B. cereus s. l.* have valuable industrial applications, such as functioning as biocontrol agents in agricultural environments [3,4,5]. However, other strains can cause food spoilage [6,7,8] and even result in serious illnesses or fatalities in both humans [1,9,10,11,12,13] and animals [14,15,16]. In the environment, *B. cereus s. l.* occurs in both vegetative and spore form [17]. Spores resist unfavorable environmental factors, including high and low temperatures, as well as limited access to water, nutrients, and radiation [18,19]. The ability of the *B. cereus* group to form heat-resistant spores is particularly critical in the food industry, as spores surviving process, e.g., pasteurization, may adversely affect the quality of the final product. Studies have shown that some strains of *B. cereus* group spores can survive passage through the stomach, depending on their growth stage, strain type, and the age of the person consuming them [20].

Illnesses caused by members of *B. cereus s. l.* can vary greatly in severity, ranging from mild to severe or even fatal. These illnesses can include anthrax and anthrax-like symptoms [15,21], as well as foodborne intoxication causing vomiting, diarrhoea, and other infections unrelated to the gastrointestinal system [22,23,24]. Emetic syndrome is caused by cereulide, which is pre-formed in food. Emetic syndrome is primarily observed in a subgroup within the phylogenetic group III of *B. cereus* [25,26,27,28]. These toxins are present in food prior to consumption and exhibit remarkable resistance to inactivation during processing. This is due to their stability even when exposed to high heat treatments such as roasting, frying, microwaving, and prolonged exposure to 121 °C for up to 2 h. Cereulide can withstand exposure to a broad range of pH levels, ranging from 2 to 10. Temperature can affect cereulide production, especially in the case of mesophilic *B. cereus* at lower temperatures. It has been reported that the largest amounts of cereulide were produced by some strains of *B. cereus* at 12 °C and 15 °C, rather than at 30 °C or 37 °C, indicating that significant toxin levels can be observed even at moderate temperatures (12 °C or 15 °C). Additionally, low temperatures play a role in the formation of isocereulide and promote a shift in the toxin composition towards isoforms with higher toxicity [29]. This toxin affects mitochondria, leading to dysfunction in various organs such as the liver, brain, and intestines. It can also affect body systems, including the immune and nervous systems. Haemolysin BL, non-hemolytic enterotoxin, and cytotoxin K are responsible for causing diarrheal syndrome [25]. The diarrheal form of *B. cereus s. l.* typically manifests 8–16 h after exposure, while the emetic form usually begins 0–5 h. Consuming food containing high levels of *B. cereus s. s.* vegetative cells or spores (>10^5^ CFU/g) can lead to food poisoning. In the small intestine, bacterial production of enterotoxins can cause symptoms like abdominal pain and diarrhea [20,25,30]. The *B. cereus* group is responsible for 1.4% to 12% of foodborne outbreaks worldwide. According to World Health Organization data, *B. cereus s. l.* is recognized as a foodborne pathogen, estimated to cause approximately 256,775 cases of foodborne illnesses worldwide each year [31].

Recently, new strains of *B. cereus s. s.* have been identified, which were traditionally considered to cause self-limiting foodborne illness [32]. However, these new strains have been linked to anthrax-like disease in mammals, including humans and livestock animals, such as cattle [33]. The strains exhibit both genotypic and phenotypic traits of *B. cereus s. s.* and *B. anthracis*. The symptoms and mortality rates of anthrax-like disease caused by these strains are those caused by *B. anthracis*. Atypical strains of *B. cereus s. s.* and *B. cereus* biovar *anthracis* have the potential to express different capsules, depending on the plasmids [32].

*B. cereus s. l.* infections are often caused by the ingestion of pre-formed toxins produced by the bacteria in contaminated food, rather than by active bacterial growth in the body; antibiotics are not necessary for the treatment of these infections. However, the extent to which *B. cereus s. l.* strains may serve as a source of transferable antibiotic resistance genes in the food chain has not been well-studied [34,35]. *B. cereus s. s.* is often resistant to β-lactam antibiotics, as well as to ciprofloxacin, cloxacillin, erythromycin, tetracycline, and streptomycin.

According to the “The European Union One Health 2022 Zoonoses Report” numerous instances of foodborne outbreaks (FBOs) linked to bacterial toxins were reported, with *B. cereus* group toxins leading the list and contributing to two fatalities in the EU [36]. In 2022, there was a notable surge in FBOs attributed to *B. cereus* group toxins compared to the previous year, with an additional 219 incidents reported, marking a relative increase of 251.7%. The substantial rise was attributed to France, which accounted for 90.8% of all FBOs associated with *B. cereus s. l.* toxins.

*B. cereus s. l.* genomes are highly conserved, with sizes of 5.2 to 5.9 Mb, except for *B. cytotoxicus*, which is the most divergent of the group, with a chromosome of 4.085 Mb [37].

Distinguishing between different species of *B. cereus s. l.* can prove challenging when using traditional methods such as phenotypic and biochemical characteristics. Consequently, alternative classification systems based on molecular typing [26,27,28] and whole-genome sequencing (WGS) techniques have been proposed [38,39,40]. The use of WGS has significant implications for food safety, particularly in monitoring foodborne diseases and investigating outbreaks [41]. Many traditional microbiological analyses are being replaced by WGS analysis for rapid identification and characterization of bacterial pathogens, including serotyping, antimicrobial resistance (AMR), and virulence profiling. The study of infectious agents and antimicrobial resistance is undergoing a transformation from traditional microbiology to genomics, adopting a “One Health” approach.

The aim of this study was to examine the genetic diversity, including toxigenic and antimicrobial resistance genes, of *B. cereus s. l.* strains isolated from ready-to-eat food products in Poland.

## 2. Materials and Methods

### 2.1. Bacterial Strains and Culture Conditions

In total, 550 presumptive *B. cereus* group strains isolated from various ready-to-eat foods, were examined in this study. The strains, collected between 2018 and 2020, were gathered by Sanitary and Epidemiological Stations as part of the national official control and monitoring program, following plans devised by the Department of Food Safety of NIPH NIH-NRI (refer to Supplementary Table S1). The food samples were tested according to PN-EN ISO 7932:2005 [42], accredited by the Polish Center of Accreditation, at the Sanitary and Epidemiological Stations. In brief, 10 g of each food sample was homogenized in 90 mL of buffered peptone water (BPW). A 0.1 mL aliquot from the initial suspension, along with subsequent decimal dilutions, was plated onto mannitol egg yolk polymyxin agar plates (MYP agar). After incubation for 24–48 h at 30 °C, the typical colonies were enumerated and subjected to the hemolysis reaction test. Presumptive *B. cereus* strains were sent to the Department of Food Safety of NIPH NIH-NRI for confirmation and characteristics (PCR, WGS). Confirmation tests for all strains were performed in accordance with PN-EN ISO 7932:2005, which included typical growth on MYP medium–large, pink colonies surrounded by a zone of turbidity (Oxoid, Basingstoke, United Kingdom) and hemolysis test on sheep blood agar (Biomaxima, Lublin, Poland) [42]. Strains were recovered from −80 °C brain heart infusion broth (BHI, Oxoid) with 20% glycerol (Merck, Darmstadt, Germany).

### 2.2. Extraction of DNA from Presumptive B. cereus Strains for PCR Reaction

DNA from 550 strains of *B. cereus* group was extracted using the chelex 100 resin (BioRad, Poland) as described before by Kowalska et al. [43].

### 2.3. B. cereus Group Identification by PCR Reaction

A modified method described by Hansen et al. [44] was used to identify the strains belonging to the *B. cereus* group. The oligonucleotides specific for *B. cereus* group strains *16S rDNA*-targeting-5′-TCG AAA TTG AAA GGC GGC-3′ and 5′-GGT GCC AGC TTA TTC AAC-3′ were used (Genomed, Warsaw). The final 25 μL of the PCR mixture included 2.5 µL DreamTaq buffer (10× concentrated, Thermo Fisher Scientific, Waltham, MA, USA), 3.75 µL of a dNTPs mix (2 mM, Thermo Fisher Scientific), 1 µL of MgCl_2_ (1.25 mM, Thermo Fisher Scientific), 1 µL of each primer (10 μM), 0.25 µL of DreamTaq polymerase (1 U, Thermo Fisher Scientific), 1 μL of DNA, and water for molecular biology (Bio-Rad, Feldkirchen, Germany). The PCR was performed under the following conditions: 95 °C for 10 min; 30 cycles of 94 °C for 15 s, 63 °C for 45 s, and 72 °C for 2 min; 72 °C for 2 min. The amplified PCR products were subsequently assessed on a 1.5% (*w*/*v*) agarose gel (Prona, Narew, Poland) in a Tris–borate–ethylenediaminetetraacetic acid (TBE) buffer (1×) with Midori Green Advance DNA Stain (Genetics, Düren, Germany). The gels were run at 120 V for 1 h. The expected PCR product size was 288 bp.

### 2.4. B. cereus Group Toxin Identification Using Multiplex PCR

The genes for nhe, hbl, cytK, and ces were PCR-amplified according to the method described by Kowalska et al. [43]. The final reaction 25 µL mixtures contained 0.25 µL of the Dream Taq polymerase (5 U, Thermo Fisher Scientific, USA), 2.5 µL of the Dream Taq buffer (10×, Thermo Fisher Scientific, USA), 2.5 µL of a dNTP mix (2 mM, Thermo Fisher Scientific, USA), 1 µL of MgCl_2_ (1.25 mM, Thermo Fisher Scientific, USA), 0.6 µL of mix oligonucleotide primers (see Table 1), 2.5 µL of the Dream Taq buffer (10× concentrated, Thermo Fisher Scientific, USA), 2.5 µL of a dNTP mix (2 mM, Thermo Fisher Scientific, USA), 1 µL of MgCl2 (1.25 mM, Thermo Fisher Scientific, USA), 0.25 µL of the Dream Taq polymerase (5 U, Thermo Fisher Scientific, USA), 1 µL of template DNA and water for molecular biology (Bio-rad, Germany). Reaction conditions were as follows: 95 °C for 15 min, cycles of 95 °C for 30 s, 49 °C for 30 s, and 72 °C for 1 min, with a final extension at 72 °C for 2 min. The amplified PCR products were analyzed on a 1.5% (*w*/*v*) agarose gel (Prona, Poland) in 1× TBE buffer containing 0.15 µg/mL of Midori Green Advance DNA Stain (Genetics, Germany). A GeneRuler™ 1 kb DNA Ladder and GeneRuler™ 100 bp DNA Ladder (both from Thermo Fisher Scientific, USA) were used as molecular weight markers. The gels were run at 120 V for 1 h and visualized using a digital imaging system.

### 2.5. Extraction of DNA for Whole-Genome Sequencing (WGS)

For in silico studies, fifty strains were selected from the toxin group, each containing at least two of the four toxins, with most strains containing three toxins. Genomic DNA from the selected *B. cereus* group was extracted using Genomic Mini AX Bacteria+ (A and A Biotechnology, Gdańsk, Poland) according to the manufacturer’s instructions. DNA concentration was determined using a NanoDrop One (Invitrogen, Thermo Fisher Scientific, Waltham, MA, USA).

### 2.6. Whole-Genome Sequencing

Genomic DNA concentration was measured before the library preparation procedure by fluorimetry using PicoGreen reagent (Life Technologies, Thermo Fisher Scientific). The measurement was performed on a Tecan Infinite device. Libraries were prepared using the NEBNext Ultra II DNA Library Prep Kit for Illumina (New England Biolabs, Ipswich, MA, USA) according to the manufacturer’s instructions. Sequencing was conducted by Genomed (Warsaw, Poland) using MiSeq paired-end (PE) technology 2 × 300 nt and the MiSeq Reagent Kit v3 (600-cycle, Illumina) according to the manufacturer’s protocol.

### 2.7. Bioinformatics Analysis

#### 2.7.1. Genome Assembly and Quality Control

Readings were filtered using Cutadapt version 3.0. Quality control of the sequencing results was performed with FastQC software version 0.12.1. De novo assembly was conducted using Spades version 3.14.5.

#### 2.7.2. In Silico Typing and Taxonomic

The assembled genome of *B. cereus s. l.* can be effectively classified into one of eight phylogenetic groups based on the scheme proposed by Carroll et al. [45]. This classification can be performed using the BTyper tool version 3.4.0 https://github.com/lmc297/BTyper3 (accessed on 6 May 2024).

In our study, BTyper tool was used to designate ANI-based species, ANI-based subspecies, and biovar assignment using a standardized nomenclatural framework for *B. cereus s. l.* The assigned type strains were calculated with ANI values between a query genome and the genomes of all published *B. cereus* group species types, reporting the type of strain that produces the highest ANI value. Additionally, taxonomic identification was performed using Ridom SeqSphere+ software version 10.0.4. Furthermore, multi-locus sequence typing (MLST, using the PubMLST *B. cereus* database at Ridom SeqSphere+), clonal complex (CC) (Ridom SeqSphere+), and cgMLST (PubMLST) were performed.

#### 2.7.3. SNP Analysis

We conducted SNP analysis using the CSI Phylogeny 1.4 tool provided by the Center for Genomic Epidemiology, accessed at www.genomicepidemiology.org (accessed on 6 May 2024). We generated phylogenetic trees based on our datasets and used our first genome as a reference. To visualize the Newick files that were generated, we utilized iTol https://itol.embl.de/ (accessed on 6 May 2024).

#### 2.7.4. In Silico Virulence, Antibiotics Resistance Genes, Plasmids Detection

The *B. cereus s. l.* genomes were screened to determine putative virulence factors (using the Btyper tool), antibiotic resistance genes (NCBI, AMRFinderPlus, Ridom SeqSphere+), pathogenicity toward the human host (PathogenFinder v1.1, CGE), and plasmids (PlasmidFinder v2.0, CGE). For antibiotic resistance genes, we used BLAST alignment > 90% of length and >90% identity to a protein in the database.

## 3. Results and Discussion

### 3.1. Prevalence of B. cereus s. l. in Ready-to-Eat Food Collected in Poland

This study retrospectively analyses *B. cereus* group isolates from ready-to-eat food collected from retail markets, as well as strains isolated from food poisoning cases. Most of the *B. cereus s. l.* strains were isolated from cake samples. Notably, the level of *B. cereus* group bacteria did not exceed the limit of 10^5^ CFU/g. Most foodborne outbreaks related to the *B. cereus* group have been associated with levels above this limit. Nevertheless, cases of emetic and diarrheal illness have been reported at lower levels of *B. cereus*, ranging from 10^3^ to 10^5^ CFU/g [24].

Information regarding the genetic characterization of *B. cereus s. l.* in confectionery products, both with and without cream, as well as cereal grains, flour, and cereal products are limited. Consequently, comparisons will currently focus on matrices with similar characteristics, such as milk and dairy products, or grain-based or RTE matrices [46].

According to our previous data, the prevalence of the *B*. *cereus* group in ready-to-eat samples in Poland during the 5-year period (2016–2020) was 0.75. The monitoring involved a total of 45,358 tests mainly in two categories of products—confectionery products with or without cream and cereal grains, flour, and cereal products. The analysis also covered samples collected from food poisoning cases or consumer complaints. We conducted another study which revealed that some strains of bacteria from the *B. cereus* group found in the country can produce toxins and exhibit drug resistance [47].

### 3.2. Reidentification of Strains Using Standardized Methods and PCR Methods

In our research, we conducted tests using both classical and PCR methods on 550 bacterial strains isolated from food that belonged to the *B. cereus* group. These tests allowed us to confirm the group membership. The PCR results showed that all strains belong to the *B. cereus* group (see Supplementary Table S1).

### 3.3. Identification of Toxin Using PCR Methods

The toxin gene profiles of 550 isolates of *B. cereus s. l.*, established using multiplex PCR, are presented in Table 2. The *nhe* gene was detected in 91.8% of the 550 isolates, while the *hbl* gene was found in 43.8% and the *cytK* gene in 46.9%. The *ces* gene had the lowest occurrence at only 4.2%. Approximately 4.2% of the *B. cereus s. l.* strains analyzed were absent of *hbl*, *nhe*, *cytK*, and *ces* genes. Previous studies on *B. cereus s. l.* strains isolated from 2004 to 2018 showed similar gene detection frequencies [47]. In this study, among 267 *B. cereus s. l.* isolates tested from food products, 95.51% were positive for at least one toxin gene. The *nhe* gene had the highest frequency at 91.39%, with *hbl* and *cytK* detected in 53.56% and 44.19% of strains, respectively. The *ces* gene occurred the least frequently, at 2.62%. Toxin genes such as *nhe* were detected in almost all *B. cereus s. l.* species, while the genes encoding *hbl* and *cytK* are variably present within *B. cereus s. l.* species [2]. The study by Fiedler et al. [34] revealed that 91.2%, 83.0%, and 37.4% of the isolates were positive for the presence of the *hbl*, *nhe*, and *cytK* toxin genes, respectively. Additionally, our research showed the presence of the *cytK* gene in 46.9% of the isolates, indicating a significant difference compared to the findings of Fiedler et al., who reported its presence in all tested isolates. Moreover, the *ces* gene was detected in our study at a significantly lower frequency, being present in only 4.2% of the isolates compared to the much higher frequency of 57.14% reported by Fiedler et al. According to Çöl et al., bacteria of the *B. cereus* group were also identified in ready-to-eat foods and pastry products in Turkey, with the presence of *nhe*, *hbl*, and *cytK* genes detected at rates of 91.9%, 56.8%, and 8.1%, respectively [48].

These data align with previous studies, indicating a consistent presence of these virulence factors across different regions and time frames.

### 3.4. Toxin Profiles

The strains were categorized into toxin profiles A-G based on a classification proposed by Ehling-Schulz et al. [49] and profiles H-I as proposed by Kowalska et al. [47]. The profiles are defined as follows for toxin gene presence: A (*hbl*, *nhe*, *cytK*), B (*nhe*, *cytK*, *ces*), C (*hbl*, *nhe*), D (*nhe*, *cytK*), E (*nhe*, *ces*), F (*nhe*), G (*cytK*), H (*hbl*), I (*hbl*, *cytK*). Additionally, a new profile, J, was identified, which encodes all four toxins (*hbl*, *nhe*, *cytK*, *ces*).

The isolates demonstrate significant diversity in their toxin profiles. All toxin profiles were present in Table 2, and the following distribution was observed: F (30%), A (26.4%), D (16.5%), C (14.7%), E (3.6%), I (2%), G (1.6%), H (0.4%), B (0.2%). Our investigation revealed the presence of the new J profile in 2 out of 550 isolates, indicating a prevalence of 0.4% in this study. Detailed results for all analyzed strains are provided in Supplementary Table S1. In the previous study conducted by Kowalska et al. [47], the predominant toxin profile identified was profile A, accounting for 31.1%, followed by profile F at 28.8%.

The variety of toxin profiles observed among the characterized strains emphasizes the necessity of adopting a diverse, strain-specific approach to toxin analysis. Concerning public health, variability in toxin profiles directly influences clinical manifestations and infection outcomes. A thorough comprehension of the pathogenicity and virulence mechanisms linked with each strain would enable prompt and effective clinical interventions.

### 3.5. Taxonomic Classification

The results of the taxonomic classification of the genomes of the *B. cereus* group have been presented in Supplementary Table S2. The analysis conclusively confirmed that 49 out of 50 isolates were associated with the *B. cereus* group. One strain remained unidentified (5086 B) despite characteristic growth on the MYP medium and the positive hemolysis test. WGS analyses did not confirm its belonging to the *B. cereus* group. WGS for species identification unveiled the existence of four distinct species within *B. cereus s. l.* Among these, 21 isolates (3988 A, 4011, 4022, 4220, 4032, 4036, 4144, 4422, 4686 B, 4753, 4818 C, 4819 A, 4865 C, 4912 A, 4913 A, 5065, 5082 A, 5084 B, 5085 D, 6008 A, 6066 A) exhibited the closest related relation (≥96%) to *B. cereus s. s.* Additionally, 25 isolates (3992 B, 3996, 4031, 4051, 4109, 4136, 4201, 4204, 4217, 4320 B, 4327, 4537 A, 4607 B, 4618 B, 4644, 4763, 4810, 5034 A, 5080 B, 5088 A, 5090 C, 6019 A, 6029, 6071, 6073A) exhibited the closest relation (≥93%) to *B. mosaicus*. Furthermore, the two strains exhibited the highest similarity (≥98%) to the *B. toyonensis* strain (4348, 5051 B), while one isolate closely resembled *B. mycoides* (5062). These findings underscore the genetic diversity within *B. cereus s. l.* and provide valuable insights into the relatedness of different isolates within *B. cereus s. l.* isolated from food. The application of Ridom Seq Sphere+ software for *B. cereus* group identification revealed the following close associations: *B. cereus s. s.* (3988 A, 3992 B, 3996 A, 4022, 4051, 4136, 4144, 4204, 4217, 4220, 4320 B, 4422, 4607 B, 4618 B, 4686 B, 4818 C, 4819 A, 5065, 5080 B, 5084 B, 5085 D, 5090 C, 6008 A, 6066 A), *B. thuringiensis* (4011, 4031, 4036, 4753, 4763, 4865 C), *B. wiedmannii* (4109, 4201, 4810, 6019 A, 6071), *B. bombysepticus* (4032, 4912 A, 4913 A, 5082 A), *B. paranthracis* (4644, 4537 A, 4327, 5088 A), *B. toyonensis* (4348, 5051 B), *B. mobilis* (6029, 6073), *B. anthracis* (5034 A), *B. mycoides* (5062), and *B. pumilus* (5086 B). Differences in identification result from the use of different taxonomic approaches. The relation between the *panC* and MLST classification of the isolates typing within the *B. cereus* group functions as a significant molecular indicator, aiding in the comprehension of genetic connections, evolution, and variety present within this bacterial cluster [45]. Based on this classification, the isolates were grouped (Supplementary Table S2). Of all the isolates, 44% were found to belong to group IV, 32% to III, 16% to II, 4% to V, 2% to VI, and 2% to unknown (Table 3). The result obtained by Didouh et al. [50] shows that the identity of the isolated strains was confirmed as *B. cereus s. l.*, and the sequencing of the *panC* gene clustered 64.7% of them in phylogenetic group III and 35.3% in group IV, which is like the results obtained in our study. These results indicated by Bogaerts et al. [35] show that all isolates originating from commercial vitamin B2 feed and food additives collected between 2016 and 2022 were classified by BTyper3 as species within the *B. cereus* group. Among the isolates, *B. mosaicus panC* clade III (*n* = 33) and *B. cereus s. s. panC* clade IV (*n* = 3) were identified.

### 3.6. Genetic Variability

Further analyses included the identification of genetic diversity and sequence types (ST) among *B. cereus s. l.* isolates. The sequences of seven housekeeping genes, analyzed using the standard MLST scheme (i.e., *glp*, *gmk*, *ilv*, *pta*, *pur*, *pyc*, and *tpi*), were examined. Among the analyzed strains, 32 ST were distinguished (ST8, ST10, ST18, ST19, ST24, ST26, ST33, ST34, ST92, ST127, ST131, ST142, ST144, ST166, ST177, ST196, ST312, ST369, ST554, ST689, ST770, ST899, ST1295, ST1329, ST1524, ST1983, ST2039, ST2264, ST2681, ST2682, ST2726, ST3239) (Table 3). Three strains could not be assigned to any known ST type. The newly identified sequence types were added to the PubMLST database using allelic codes from the seven housekeeping genes and the MLST database reference. Three previously unreported STs were identified: 3297, 3298, 3299 (Table 4).

In our study, six clonal complexes (CC) were identified: CC8 (4011), CC18 (4032, 4912 A, 4913 A, 5082 A), CC111 (5062), CC142 (4686 B, 5084 B), CC196 (5065), CC205 (5088 A). There is a lack of data in the literature regarding the occurrence of MLST types in food products such as bakery items.

*B. cereus s. s.* isolates associated with emetic food poisoning are generally linked with ST26. In our study, out of four of the five isolates belonging to ST26, three contained the *ces* gene, supporting the hypothesis that this genotype often harbors potential emetic strains [51]. However, these four strains have the *nhe* cluster gene but not *hbl*, as previously reported [52]. Additionally, we identified further sequence types: ST8, ST92, ST166, that may be significant in identifying potentially emetic strains due to the presence of the *ces* gene. Moreover, the ST166 sequence type contained all the toxin-encoding genes (*hbl*, *nhe*, *ces*, *cytK*), which makes this type potentially pose the greatest health risk.

**Figure 1:**
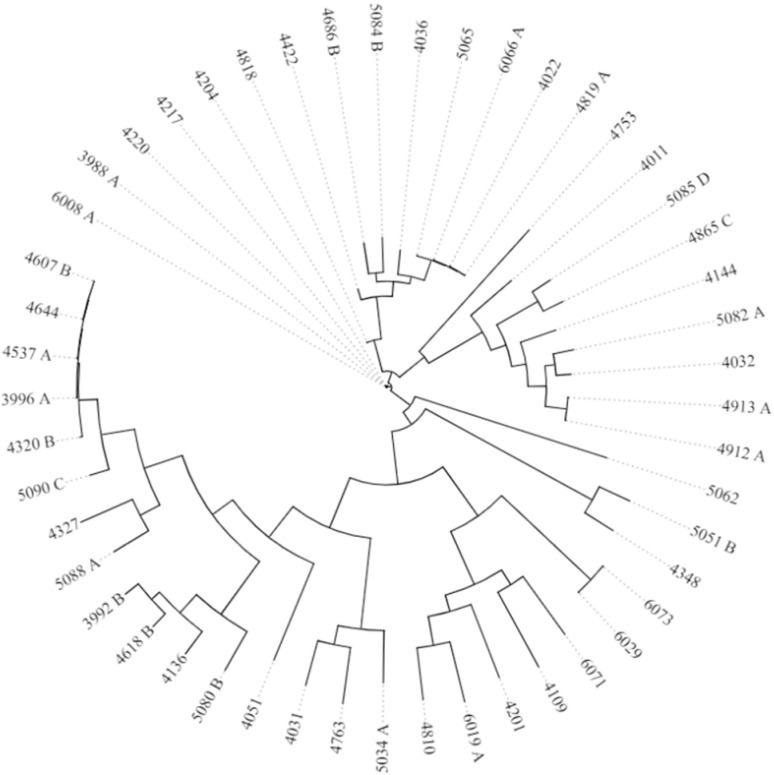
SNP phylogeny of *B. cereus* group. The CSI Phylogeny 1.4 tool provided by the Center for Genomic Epidemiology, accessed at www.genomicepidemiology.org (accessed on 6 May 2024). We generated phylogenetic trees based on our datasets and used our first genome as a reference. To visualize the Newick files that were generated, we utilized iTol (https://itol.embl.de/ (accessed on 6 May 2024)).

The findings regarding the small pairwise SNP (single nucleotide polymorphisms) distances between isolates 6008A and 3988, as well as between 6029 and 6073, are particularly interesting within the context of *B. cereus* and its presence in food products. In bacterial genomics, SNPs provide valuable insights into the genetic variation and evolutionary relationships between different strains or isolates. The close genetic relationship indicated by the small SNP differences (ranging from 5 to 12 SNPs) suggests that these isolates likely share a recent common ancestor. The visualized SNP results are presented in Figure 1.

Using the PubMLST platform, cgMLST analysis was conducted on all strains under study. The analyses revealed no discernible connections between strains isolated from food sources. cgMLST profiling was completed for 26 out of 49 *B. cereus s. l.* strains.

The analysis categorized the examined strains of *B. cereus s. l.* into the following cgSTs: 41, 56, 137, 202, 241, 246 (2 isolates), 274, 309, 383, 540, 707, 750, 774, 1715, 1721, 1796, 2526 (3 isolates), 2529, 3402, 3403, 3414, and 3491 (2 isolates).

Retrospective cgMLST analyses allow for understanding how bacterial strains have spread in the past, which can help in identifying the sources of infection outbreaks and their transmission. The results of retrospective analyses can be used to develop preventive strategies based on food monitoring.

### 3.7. Virulence Factors

Predicted phenotypes of *B. cereus s. l.* strains sequenced in this study (*n* = 49) are presented in Table 3. In eight isolates, the *ces* gene cluster responsible for producing the emetic toxin cereulide were found. The biosynthesis of cereulide is controlled by the non-ribosomal peptide synthetase (NRPS) found in *ces* gene clusters. These clusters contain the *cesA*, *cesB*, *cesC*, *cesD*, *cesH*, *cesP*, and *cesT* genes and are situated on a 270 kb megaplasmid named pCER270 (or pCERE01) [53]. These genes were identified in 8 out of 49 analyzed strains (16%), with 5 isolates belonging to clade IV and 3 to clade III. The examined isolates did not exhibit the presence of the anthrax toxin genes *cya*, *lef*, and *pagA*.

The non-hemolytic enterotoxin Nhe is composed of three elements: NheA (41 kDa), NheB (39 kDa), and NheC (105 kDa). These components are encoded by the genes *nheA*, *nheB*, and *nheC*, as indicated in studies by Dietrich et al. [30]. Among the 49 *B. cereus* group sequences examined, 48 (98%) exhibited the presence of all three genes associated with non-hemolytic enterotoxin Nhe synthesis. One strain found two from three genes (*nheA*, *nheB*). The occurrence of the toxin gene cluster was found in all clades (II, III, IV, V, VI).

Hemolysin BL is composed of binding protein B (37 kDa), which is coded by the *hblA* gene, and two lytic components: L1 (38 kDa), which is encoded by *hblC*, and L2 (46 kDa), encoded by *hblD*. Together, these genes form the hblCDA operon. Additionally, certain strains of the *B. cereus* group also possess the *hblB* gene, classified as a pseudogene, within the *hblCDAB* operon [30,54,55]. The presence of all genes in cluster HBL toxin genes was demonstrated in 53% of the analyzed strains (26/49). In one strain, only *hblC* and *hblD* were found, while another strain had *hblA* and *hblB*, and a third strain contained three out of the four genes: *hblA*, *hblB*, and *hblD*. The diarrheal toxin genes (*hblA-D*) were absent in 21 out of the 49 strains examined. The toxin gene cluster was found in clades II (5 strains), III (2), IV (18), V (3), and VI (1).

The single-protein cytotoxin (CytK), with cytotoxic, dermonecrotic, and hemolytic activity, is encoded by the *cytK* gene and is responsible for causing diarrheal-type infections [56]. The authors identified two variants of the *cytK* gene: *cytK-1* (the original *cytK*) and *cytK-2.* Although not all CytK-2 toxins may be as harmful as CytK-1, which was originally isolated, CytK-2 toxins still contribute to enterotoxicity [57]. The *cytK-2* was found in 26 out of 49 (52%) of isolates. The isolates were classified into clades III (5 strains), IV (20), and V (1).

The findings reported by Bianco et al. revealed that among 139 isolates obtained from milk and cheeses samples, the *nheABC* genes cluster encoding the non-hemolytic toxin was detected in 90% of the isolates [58]. Most of these isolates contained the entire gene cluster, with only three exceptions. Additionally, the *hblCDA* gene cluster was identified in 61% of the isolates, with the *hblA*, *hblC*, and *hblD* genes present at frequencies of 68%, 64%, and 75%, respectively. The *cytK* gene was identified in 44% of the isolates, while the *ces* gene was detected in 8% of the total isolates. In comparison to our study, the *nheABC* gene cluster was observed in 98% of the isolates, suggesting a slightly higher occurrence than reported by Bianco et al. [58]. Similar results were found for the presence of the HBL toxin genes in both studies. Moreover, our study identified various combinations of genes within the HBL cluster, indicating the genetic diversity of this toxin group. The frequency of the *cytK* gene was also comparable between the Bianco et al. [58] study (44%) and ours (52%). Notably, both studies reported a relatively low prevalence of the *cesB* gene.

The prevalence of *nheABC*, *hblACD*, *cytK*, and *cesB* was examined in 54 *B. cereus s. s.* isolates collected from dairy products between 2018–2019 in China [59]. Detection rates for *hblA*, *hblC*, and *hblD* were 57.4%, 68.5%, and 16.7%, respectively, with the *hblACD* gene cluster present in 11.1% of the isolates. In contrast, nearly all isolates (94.4%) contained *nheABC* genes, with only 5.6% lacking either *nheA* or *nheB* genes. Most isolates (75.9%) also carried the *cytK* gene. However, the *cesB* gene was detected in only 11.1% of the strains, significantly lower than the detection rates for the other ten diarrheal enterotoxin genes analyzed.

Overall, the results indicate significant diversity in toxin gene profiles among *B. cereus s. l.* strains, with important implications for understanding their pathogenic potential and associated public health risks. The consistency between WGS and PCR results further validates the accuracy of the analytical methods employed in this study.

Another toxin produced by this group is sphingomyelinase. Sphingomyelinase belongs to a group of bacterial extracellular toxins with a molecular mass of 34 kDa and exhibits potent hemolytic activity against sphingomyelin-rich erythrocytes in mammals (eg. ruminants) [60]. In *B. cereus s. l.*, sphingomyelinase is a virulence factor associated with septicemia. The sphingomyelinase gene (*sph*) was detected in nearly 98% of the isolates across all clades. Similar findings were reported by Didouh et al. [50], where the *sph* gene was detected in 88.2% of isolates of milk containing pasteurized, unpasteurized local raw cow and recombined milk.

Some atypical *B. cereus s. s.* can produce a unique exopolysaccharide (Bps) capsule that is not found in *B. anthracis* or *B. cereus* biovar *anthracis* strains [32]. This capsule is encoded by a nine-gene operon, *bpsX-H*, present on the plasmid pBC210. *B. cereus* biovar *anthracis* strains do not carry the pBC210 plasmid but possess pBCXO2 plasmid, which is highly similar to the pXO2 found in *B. anthracis*. The *capBCA* genes, which are responsible for the expression of the polyglutamate capsule necessary for full virulence in *B. anthracis*, are also encoded on pBC210. In this study, the exopolysaccharide capsule gene (*bpsE*) was detected in all strains analyzed, while the *bpsH* gene was identified in 25 out of 49 strains, and the *bpsD* gene in two strains.

Studies have shown the presence of plasmids pBT9727 in strains 6029, 4819 A, 4644, and 4607 B, originating from the pathogenic *B. thuringiensis* serovar *konkukian* strain 97–27 [61]. The pBT9727 in strain 6029 exhibits similarities to the *B. anthracis* pXO2 plasmid (GeneBank CP_000047) [62]. Analysis of the predicted coding regions of both plasmids revealed that pBT9727 shares 89% (82 out of 92) of its presumed coding sequences with pXO2. Additionally, the occurrence of plasmids pBMB67 was detected in strain 4753, which is involved in cell-to-cell signaling and may play a role in regulating various cellular processes, possibly including exoprotease production [63]. Furthermore, plasmid pBMB165 was identified in strain 6066 A, which encodes a hypothetical protein, Rep165, and replication-associated protein genes [64]. pBMB165 is associated with the spore–crystal association (SCA) phenotype in *B. thuringiensis* [65].

### 3.8. Antibiotic Resistance Genes

Considering the emergence of antibiotic-resistant *B. cereus* group strains, which can lead to treatment failures, raising awareness of this issue has become crucial for public health. We identified genes potentially associated with resistance to several antibiotics: beta-lactam, fosfomycin, vancomycin, erythromycin, telithromycin, tylosin, chloramphenicol, streptothricin, tetracycline (Table 5). Among the isolates, 100% harbored genes linked to beta-lactam resistance: 45 out of 49 carried the *bla*-BcII family subclass B1 metallo-beta-lactamase gene, which is associated with carbapenem resistance; 36 out of 49 had the *bla* class A beta-lactamase *bla1* gene; 4 out of 49 carried the *blaTEM-181* gene, a class A beta-lactamase; and 1 out of 49 contained a *blaTEM* gene from the TEM family of class A beta-lactamases. This study also confirmed the presence of fosfomycin resistance genes in 65% of isolates (32/49) carrying the *fosB* gene (from the FosB/FosD family of fosfomycin resistance bacillithiol transferases) and in six isolates carrying *fosBx1*. Other resistance genes were found less frequently, including *vanR-A* (VanA-type vancomycin resistance DNA-binding response regulator), which was present in 8% of the isolates and is associated with vancomycin resistance; *satA* (streptothricin N-acetyltransferase), linked to streptothricin resistance, was found in 6% of isolates; and *tet* (45) (tetracycline efflux MFS transporter), responsible for tetracycline resistance, was detected in 4% of the isolates. Resistance to macrolide antibiotics, through the *mphL* gene (macrolide 2′-phosphotransferase), was identified in one isolate [66].

The presence of mobile genetic elements in *B. cereus s. l.*, such as transposons and plasmids, facilitate the acquisition and transfer of resistance genes from the environment. This contributes to the emergence of a new resistance phenomenon, characterized by elevated levels of resistance, particularly to multiple drugs. While antibiotic prophylaxis may not be critical in the context of food poisoning caused by this microorganism, in other cases (e.g., blood infections), the antimicrobial resistance of the *B. cereus* group poses a significant public health risk [34].

Typically, *B. cereus s. s.* is resistant to penicillin G and other beta-lactam antibiotics as noted in both in silico analyses and previous studies [34,67]. Resistance to beta-lactam antibiotics, conducted by the *bla* or *bla2* gene, is frequently observed in *B. cereus s. s.* Additionally, acquired resistance to other antibiotics, such as tetracycline, ciprofloxacin, amikacin, and chloramphenicol, has been reported [34]. Osama et al. found that *B. cereus s. l.* strains isolated from Egyptian dairy products showed 2.6% resistance to tetracycline, 5.6% resistance to erythromycin, 67.9% resistance to streptomycin, and 100% to colistin [68]. Similarly, Zhai et al. confirmed *B. cereus* resistance to tetracycline and erythromycin of *B. cereus* [69]. Antimicrobials such as vancomycin, gentamicin, chloramphenicol, and carbapenems are commonly prescribed for treating nosocomial *B. cereus s. s.* bloodstream infections due to their proven antimicrobial efficacy [70,71]. However, resistance to vancomycin, linked to the *vanR-A* (VanA-type vancomycin resistance DNA-binding response regulator VanR) gene, was observed in only 8% of isolates, which contrasts with the study by Fraccalvieri et al., where all 51 ice cream samples contained strains carrying genes conferring resistance to beta-lactam and fosfomycin antibiotics [72].

## 4. Conclusions

This study provides a comprehensive retrospective analysis of *B. cereus* group isolates from ready-to-eat foods collected between 2018 and 2020, including strains linked to food poisoning cases. The majority of *B. cereus s. l.* strains came from ready-to-eat cake samples. The research reveals diverse toxin profiles among the examined strains, emphasizing the need for a strain-specific approach in toxin analysis. Whole-genome sequencing confirmed the genetic diversity within the *B. cereus s. l.*, identifying distinct species and clonal complexes. This study also investigated the prevalence of antibiotic resistance genes, highlighting potential concerns for public health. Overall, this research enhances our understanding of *B. cereus* group diversity, toxin production, and antibiotic resistance in food-related contexts, underlining the importance of continued monitoring and research in this field.

## Figures and Tables

**Table 1:** Primers used in the multiplex PCR for detecting toxin genes in *B. cereus s. l*.

Primer Name	Sequence (5′–3′)	Final Concentration of the Primers (mM)	Targeted Gene	Product Size (bp)
cytK-F	ACA GAT ATC GGI CAA AAT GC	200	*cytK*	421
cytK-R	CAA GTI ACT TGA CCI GTT GC	200		
Nhe-F	AAG CIG CTC TTC GIA TTC	150	*nhe*	766
Nhe-R	ITI GTT GAA ATA AGC TGT GG	150		
Hbl-F	GTA AAT TAI GAT GAI CAA TTT C	500	*hbl*	1091
Hbl-R	AGA ATA GGC ATT CAT AGA TT	500		
Ces-F	GGT GAC ACA TTA TCA TAT AAG GTG	100	*ces*	1271
Ces-R	GTA AGC GAA CCT GTC TGT AAC AAC A	100		

**Table 2:** The toxin profile of *Bacillus cereus* group isolated from retail food in Poland.

Profile	Genes				Source	
					(Number of Isolates)	
	*hbl*	*nhe*	*cytK*	*ces*	Pastries (523)	Mix Products from Food Poisoning (27)
A					137	8
B					1	
C					74	7
D					90	1
E					18	2
F					161	4
G					9	
H					2	
I					8	3
J *					2	
None detected					21	2

* New classification toxin profile; black boxes—gene presence, white boxes—gene absence. The source of strain isolation from food products and detailed results are described in Supplementary Table S1.

**Table 3:** MLST, CC, and virulence of *B. cereus s. l.* strains sequenced in this study (*n* = 49).

Strain Number	MLST	CC	cgMLST	Virulence																										
				Anthrax Toxin (Genes)			Emetic Toxin Cereulide (Genes)				Diarrheal Toxin Nhe (Genes)			Diarrheal Toxin Hbl (Genes)				Diarrheal Toxin CytK (Genes)	Sphingomyelinase (Gene)	Capsule (Genes)		Exopolysaccharide Bps (Genes)								
				*cya*	Lef	*pagA*	*cesA*	*cesB*	*cesC*	*cesD*	*nheA*	*nheB*	*nheC*	*hblA*	*hblB*	*hblC*	*hblD*	*cytK2*	*Sph*	*cap*	*has*	*bpsX*	*bpsA*	*bpsB*	*bpsC*	*bpsD*	*bpsE*	*bpsF*	*bpsG*	*bpsH*
3988 A	34	−	3491	−	−	−	−	−	−	−	+	+	+	−	−	−	−	+	+	−	−	−	−	−	−	−	*+*	−	−	*+*
3992B	2681	−	137	−	−	−	−	−	−	−	+	+	+	−	−	−	−	−	+	−	−	−	−	−	−	−	*+*	−	−	*+*
3996 A	26	−	309	−	−	−	−	−	−	−	+	+	−	−	−	−	−	+	+	−	−	−	−	−	−	−	*+*	−	−	*−*
4011	8	8	56	−	−	−	+	+	+	+	+	+	+	−	−	−	−	−	+	−	−	−	−	−	−	−	*+*	−	−	*−*
4022	24	−	2526	−	−	−	−	−	−	−	+	+	+	+	+	+	+	+	+	−	−	−	−	−	−	−	*+*	−	−	*−*
4031	2264	−	−	−	−	−	−	−	−	−	+	+	+	+	+	+	+	+	+	−	−	−	−	−	−	−	*+*	−	−	*+*
4032	18	18	774	−	−	−	−	−	−	−	+	+	+	+	+	+	+	+	+	−	−	−	−	−	−	−	*+*	−	−	*+*
4036	19	−	−	−	−	−	−	−	−	−	+	+	+	+	+	+	+	+	+	−	−	−	−	−	−	*+*	*+*	−	−	*+*
4051	369	−	−	−	−	−	−	−	−	−	+	+	+	−	−	−	−	−	+	−	−	−	−	−	−	−	*+*	−	−	*−*
4109	131	−	−	−	−	−	−	−	−	−	+	+	+	−	−	+	+	−	+	−	−	−	−	−	−	−	*+*	−	−	*+*
4136	92	−	−	−	−	−	+	+	+	+	+	+	+	−	−	−	−	−	+	−	−	−	−	−	−	−	*+*	−	−	*+*
4144	3297	−	−	−	−	−	−	−	−	−	+	+	+	+	+	+	+	+	+	−	−	−	−	−	−	−	*+*	−	−	*−*
4201	689	−	−	−	−	−	−	−	−	−	+	+	+	+	+	+	+	−	+	−	−	−	−	−	−	−	*+*	−	−	*−*
4204	166	−	−	−	−	−	+	+	+	+	+	+	+	+	+	+	+	+	+	−	−	−	−	−	−	*+*	*+*	−	−	*+*
4217	NA	−	−	−	−	−	+	+	+	+	+	+	+	+	+	−	−	+	+	−	−	−	−	−	−	−	*+*	−	−	*−*
4220	1524	−	1721	−	−	−	−	−	−	−	+	+	+	+	+	+	+	+	+	−	−	−	−	−	−	−	*+*	−	−	*+*
4320 B	26	−	202	−	−	−	+	+	+	+	+	+	+	−	−	−	−	−	+	−	−	−	−	−	−	−	*+*	−	−	*−*
4327	770	−	−	−	−	−	−	−	−	−	+	+	+	−	−	−	−	+	+	−	−	−	−	−	−	−	*+*	−	−	*−*
4348	3299	−	−	−	−	−	−	−	−	−	+	+	+	+	+	+	+	−	+	−	−	−	−	−	−	−	*+*	−	−	*+*
4422	177	−	1715	−	−	−	−	−	−	−	+	+	+	+	+	+	+	+	+	−	−	−	−	−	−	−	*+*	−	−	*+*
4537 A	26	−	707	−	−	−	+	+	+	+	+	+	+	−	−	−	−	−	+	−	−	−	−	−	−	−	*+*	−	−	*−*
4607 B	26	−	274	−	−	−	+	+	+	+	+	+	+	−	−	−	−	−	+	−	−	−	−	−	−	−	*+*	−	−	*−*
4618 B	127	−	3403	−	−	−	−	−	−	−	+	+	+	−	−	−	−	−	+	−	−	−	−	−	−	−	*+*	−	−	*+*
4644	26	−	2529	−	−	−	+	+	+	+	+	+	+	−	−	−	−	−	+	−	−	−	−	−	−	−	*+*	−	−	*−*
4686 B	1329	142	−	−	−	−	−	−	−	−	+	+	+	+	+	+	+	+	+	−	−	−	−	−	−	−	*+*	−	−	*−*
4753	10	−	241	−	−	−	−	−	−	−	+	+	+	+	+	+	+	+	+	−	−	−	−	−	−	−	*+*	−	−	*−*
4763	3298	−	−	−	−	−	−	−	−	−	+	+	+	−	−	−	−	−	+	−	−	−	−	−	−	−	*+*	−	−	*−*
4810	312	−	−	−	−	−	−	−	−	−	+	+	+	+	+	+	+	−	+	−	−	−	−	−	−	−	*+*	−	−	*−*
4818 C	177	−	3402	−	−	−	−	−	−	−	+	+	+	+	+	−	+	+	+	−	−	−	−	−	−	−	*+*	−	−	*+*
4819 A	24	−	2526	−	−	−	−	−	−	−	+	+	+	+	+	+	+	+	+	−	−	−	−	−	−	−	*+*	−	−	*+*
4865 C	33	−	1796	−	−	−	−	−	−	−	+	+	+	+	+	+	+	+	+	−	−	−	−	−	−	−	*+*	−	−	*−*
4912 A	18	18	−	−	−	−	−	−	−	−	+	+	+	+	+	+	+	+	+	−	−	−	−	−	−	−	*+*	−	−	*−*
4913 A	18	18	−	−	−	−	−	−	−	−	+	+	+	+	+	+	+	+	+	−	−	−	−	−	−	−	*+*	−	−	*−*
5034 A	1983	−	−	−	−	−	−	−	−	−	+	+	+	−	−	−	−	+	+	−	−	−	−	−	−	−	*+*	−	−	*+*
5051 B	24	−	−	−	−	−	−	−	−	−	+	+	+	+	+	+	+	+	+	−	−	−	−	−	−	−	*+*	−	−	*+*
5062	554	111	−	−	−	−	−	−	−	−	+	+	+	+	+	+	+	−	+	−	−	−	−	−	−	−	*+*	−	−	*−*
5065	196	196	383	−	−	−	−	−	−	−	+	+	+	+	+	+	+	−	+	−	−	−	−	−	−	−	*+*	−	−	*−*
5080 B	1295	−	540	−	−	−	−	−	−	−	+	+	+	−	−	−	−	+	+	−	−	−	−	−	−	−	*+*	−	−	*−*
5082 A	18	18	−	−	−	−	−	−	−	−	+	+	+	+	+	+	+	+	+	−	−	−	−	−	−	−	*+*	−	−	*−*
5084 B	142	142	41	−	−	−	−	−	−	−	+	+	+	+	+	+	+	+	+	−	−	−	−	−	−	−	*+*	−	−	*+*
5085 D	3239	−	−	−	−	−	−	−	−	−	+	+	+	+	+	+	+	−	+	−	−	−	−	−	−	−	*+*	−	−	*+*
5088 A	2726	205	750	−	−	−	−	−	−	−	+	+	+	−	−	−	−	−	+	−	−	−	−	−	−	−	*+*	−	−	*−*
5090 C	144	−	3414	−	−	−	−	−	−	−	+	+	+	−	−	−	−	−	+	−	−	−	−	−	−	−	*+*	−	−	*−*
6008 A	34	−	3491	−	−	−	−	−	−	−	+	+	+	−	−	−	−	+	+	−	−	−	−	−	−	−	*+*	−	−	*+*
6019 A	2039	−	−	−	−	−	−	−	−	−	+	+	+	+	+	+	+	−	+	−	−	−	−	−	−	−	*+*	−	−	*+*
6029	2682	−	246	−	−	−	−	−	−	−	+	+	+	−	−	−	−	−	+	−	−	−	−	−	−	−	*+*	−	−	*+*
6066 A	24	−	2526	−	−	−	−	−	−	−	+	+	+	+	+	+	+	+	+	−	−	−	−	−	−	−	*+*	−	−	*+*
6071	899	−	−	−	−	−	−	−	−	−	+	+	+	+	+	+	+	−	+	−	−	−	−	−	−	−	*+*	−	−	*−*
6073	2682	−	246	−	−	−	−	−	−	−	+	+	+	−	−	−	−	−	+	−	−	−	−	−	−	−	*+*	−	−	*+*

CC/cgMLST (−) not designated (+) presence of the gene; (−) absence of the genes.

**Table 4:** The newly identified sequence types (STs) of *B. cereus s. l.* scheme.

Strain	Glp	Gmk	Ilv	Pta	Pur	Pyc	Tpi	MLST
4144	37	9	151	18	12	14	7	3297
4348	87	26	78	186	91	264	30	3299
4763	34	1	32	16	18	37	24	3298

**Table 5:** Antibiotic resistance genes of *B. cereus s.l* strains sequenced in this study (*n* = 49).

Strain Number	Beta-Lactam				Fosfomycin		Vancomycin	Erythromycin	Telithromycin	Tylosin	Chloramphenicol	Streptothricin	Tetracycline
	*bla*	*blaTEM*	*blaTEM-181*	*bla2*	*fosB*	*fosBx1*	*vanR-A*	*mphL*	*mphL*	*mphL*	*catA1*	*satA*	*tet (45)*
3988 a	+	−	−	+	+	−	−	−	−	−	−	−	−
3992 b	−	−	−	+	−	−	−	−	−	−	−	+	−
3996 a	+	−	−	+	+	−	−	−	−	−	−	−	−
4011	+	−	−	+	+	−	−	−	−	−	−	−	−
4022	+	−	−	+	−	+	−	−	−	−	−	−	−
4031	+	−	−	−	+	−	−	−	−	−	−	−	−
4032	+	−	−	+	+	−	−	−	−	−	−	−	−
4036	+	−	−	+	−	+	−	−	−	−	−	−	−
4051	−	−	−	+	+	−	−	−	−	−	−	−	−
4109	+	−	−	−	+	−	−	−	−	−	−	−	−
4136	−	−	−	+	−	−	−	−	−	−	−	+	−
4144	+	−	−	+	+	−	−	−	−	−	−	−	−
4201	+	−	−	+	−	−	−	−	−	−	−	−	−
4204	+	−	−	+	+	−	−	−	−	−	−	−	−
4217	+	−	−	+	+	−	−	−	−	−	−	−	−
4220	+	−	−	+	−	+	−	−	−	−	−	+	−
4320 B	+	−	−	+	+	−	−	−	−	−	−	−	−
4327	−	−	−	+	−	−	−	−	−	−	−	−	−
4348	+	+	−	−	−	−	−	−	−	−	−	−	−
4422	+	−	−	+	+	−	−	−	−	−	−	−	−
4537 A	+	−	−	+	+	−	−	−	−	−	−	−	−
4607 B	+	−	−	+	+	−	−	−	−	−	−	−	−
4618 B	−	−	−	+	−	−	−	−	−	−	−	−	−
4644	+	−	−	+	+	−	−	−	−	−	−	−	−
4686 B	+	−	+	+	+	−	−	−	−	−	+	−	−
4753	+	−	−	+	+	−	−	−	−	−	−	−	−
4763	+	−	−	+	−	−	+	−	−	−	−	−	−
4810	+	−	+	−	+	−	−	−	−	−	−	−	−
4818 C	+	−	−	+	+	−	−	−	−	−	−	−	−
4819 A	+	−	−	+	−	+	−	−	−	−	−	−	−
4865 C	+	−	+	+	+	−	+	−	−	−	−	−	+
4912 A	+	−	−	+	+	−	−	−	−	−	−	−	−
4913 A	+	−	+	+	+	−	−	−	−	−	−	−	−
5034 A	+	−	−	+	−	−	−	−	−	−	−	−	−
5051 B	+	−	−	+	−	−	−	+	+	+	−	−	−
5062	+	−	−	+	+	−	−	−	−	−	−	−	−
5065	+	−	−	+	−	+	−	−	−	−	−	−	−
5080 B	+	−	−	+	−	−	−	−	−	−	−	−	−
5082 A	+	−	−	+	+	−	+	−	−	−	−	−	−
5084 B	+	−	−	+	+	−	−	−	−	−	−	−	−
5085 D	+	−	−	+	+	−	−	−	−	−	−	−	−
5088 A	+	−	−	+	+	−	−	−	−	−	−	−	−
5090 C	+	−	−	+	+	−	−	−	−	−	−	−	−
6008 A	+	−	−	+	+	−	−	−	−	−	−	−	−
6019 A	−	−	−	+	+	−	−	−	−	−	−	−	−
6029	−	−	−	+	+	−	−	−	−	−	−	−	−
6066 A	+	−	−	+	−	+	−	−	−	−	−	−	−
6071	+	−	−	+	−	−	+	−	−	−	−	−	+
6073	−	−	−	+	+	−	−	−	−	−	−	−	−

(+) presence of the gene; (−) absence of the genes.

## Data Availability

The original contributions presented in this study are included in the article/Supplementary Materials; further inquiries can be directed to the corresponding author.

## Supplementary Materials

The following supporting information can be downloaded at: https://www.mdpi.com/article/10.3390/foods13203266/s1, Table S1: Detailed information about 550 isolates *B. cereus* group; Table S2: Taxonomic classification of isolates from RTE food (*n* = 50).
