# Supplementary material for: Characterization of the Bacillus cereus Group Isolated from Ready-to-Eat Foods in Poland by Whole-Genome Sequencing
Source: Foods. 2024 Oct 14;13(20):3266. doi: 10.3390/foods13203266 (PMC11506886; doi:10.3390/foods13203266)
Supplement: Supplementary file 1 [file foods-13-03266-s001.zip › foods-3226658-supplementary.pdf]

Supplementary Table S1. Detailed information about 550 isolates *B. cereus* group.

	Strain number	Source of isolation (* oubreak)	Year of isolation	MYP agar (typical +, atypical -)	Hemolysis (positive +, negative -)	PCR					Toxin
						B. cereus group 298 bp	*hbl* 1091 bp	*nhe* 766 bp	*cytK* 421 bp	*ces* 1271 bp	profile (A-J)
1	3987 A	Cake with non-heat-treated cream	2018	■	■	■		■	■		D
2	3987 B	Cake with non-heat-treated cream	2018	■	■	■		■	■		D
3	3988 A	Cake with non-heat-treated cream	2018	■	■	■		■	■		D
4	3988 B	Cake with non-heat-treated cream	2018	■	■	■			■		G
5	3989 A	Cake with non-heat-treated cream	2018	■	■	■		■	■		D
6	3989 B	Cake with non-heat-treated cream	2018	■	■	■		■	■		D
7	3990 A	Cake with non-heat-treated cream	2018	■	■	■		■	■		D
8	3990 B	Cake with non-heat-treated cream	2018	■	■	■		■	■		D
9	3991 A	Cake with non-heat-treated cream	2018	■	■	■		■	■		D
10	3991 B	Cake with non-heat-treated cream	2018	■	■	■		■	■		D
11	3992 A	Cake with non-heat-treated cream	2018	■	■	■		■			F
12	3992B	Cake with non-heat-treated cream	2018	■	■	■		■			F
13	3993 A	Cake with non-heat-treated cream	2018	■	■	■		■			F
14	3993 B	Cake with non-heat-treated cream	2018	■	■	■		■			F
15	3995 A	Cake with non-heat-treated cream	2018	■	■	■		■	■		D
16	3995 B	Cake with non-heat-treated cream	2018	■	■	■		■			F
17	3996 A	Cake with non-heat-treated cream	2018	■	■	■			■		G
18	3996 B	Cake with non-heat-treated cream	2018	■	■	■		■	■		D
19	4011	Alpine cake	2018	■	■	■		■		■	E
20	4012	Alpine cake	2018	■	■	■	■	■	■		A
21	4017	Polish chocolate cream cake	2018	■	■	■		■			F
22	4018	Polish chocolate cream cake	2018	■	■	■		■			F
23	4019	Polish chocolate cream cake	2018	■	■	■		■			F
24	4020	Polish chocolate cream cake	2018	■	■	■		■			F

25	4021	Polish chocolate cream cake	2018	■	■	■		■			F
26	4022	Coconut cake	2018	■	■	■	■	■	■		A
27	4023	Coconut cake	2018	■	■	■		■		■	E
28	4024	Coconut cake	2018	■	■	■		■	■		D
29	4025	Coconut cake	2018	■	■	■	■	■	■		A
30	4026	Coconut cake	2018	■	■	■		■	■		D
31	4027	Cake with cream and apples	2018	■	■	■		■			F
32	4028	Cake with cream and apples	2018	■	■	■			■		G
33	4029	Cake with cream and apples	2018	■	■	■		■			F
34	4030	Mrs Walewska cake	2018	■	■	■		■			F
35	4031	Cake with cream and apples	2018	■	■	■	■	■	■		A
36	4032	Mrs Walewska cake	2018	■	■	■	■	■	■		A
37	4033	Mrs Walewska cake	2018	■	■	■		■			F
38	4034	Mrs Walewska cake	2018	■	■	■		■			F
39	4035	Cake with not heat-treated cream	2018	■	■	■	■	■	■		A
40	4036	Cake with not heat-treated cream	2018	■	■	■	■	■	■		A
41	4037	Cake with not heat-treated cream	2018	■	■	■	■	■	■		A
42	4038	Cake with not heat-treated cream	2018	■	■	■	■	■	■		A
43	4039	Cake with not heat-treated cream	2018	■	■	■	■	■			C
44	4046	Karpatka Eclair Cake	2018	■	■	■		■			F
45	4051	Strawberry cake	2018	■	■	■		■			F
46	4052	Strawberry cake	2018	■	■	■		■			F
47	4053	Strawberry cake	2018	■	■	■		■			F
48	4054	Cake with cream	2018	■	■	■	■	■	■		A
49	4056	Cake with cream	2018	■	■	■	■	■	■		A
50	4057	Cake with cream	2018	■	■	■	■	■	■		A
51	4058	Cake with cream	2018	■	■	■	■	■	■		A
52	4086	Meter-long cake	2018	■	■	■		■			F
53	4109	Cake with cream	2018	■	■	■	■	■			C
54	4110	Cake with cream	2018	■	■	■	■	■	■		A

55	4135	Cake with whipped cream	2018								C
56	4136	Sponge-fat cake with red currant gel and toffee-flavored cream.	2018								E
57	4137	Sponge-fat cake with red currant gel and toffee-flavored cream.	2018								F
58	4138	Sponge-fat cake with red currant gel and toffee-flavored cream.	2018								F
59	4139	Sponge-fat cake with red currant gel and toffee-flavored cream.	2018								F
60	4141	Peach cake	2018								D
61	4142	Peach cake	2018								A
62	4143	Peach cake	2018								D
63	4144	Peach cake	2018								A
64	4145	Caramel cake with peanuts	2018								F
65	4146	Caramel cake with peanuts	2018								F
66	4147	Caramel cake with peanuts	2018								F
67	4148	Caramel cake with peanuts	2018								F
68	4149	Caramel cake with peanuts	2018								F
69	4150	Coffee cake	2018								F
70	4151	Coffee cake	2018								F
71	4152	Coffee cake	2018								F
72	4153	Coffee cake	2018								F
73	4156	Sponge cake with puddings	2018								D
74	4157	Sponge cake with puddings	2018								C
75	4158	Sponge cake with puddings	2018								F
76	4159	Sponge cake with puddings	2018								F
77	4160	Apple pie	2018								A
78	4161	Cake	2018								A
79	4201	Cream roulade with non-heat-treated cream	2018								C
80	4204	Pear cake with non-heat-treated cream	2018								J
81	4205	Napoleon Cake	2018								D
82	4209	Polish chocolate cream cake	2018								A

83	4216	Tomato dip*	2018	■	■	■	■	■			C
84	4217	Cake with nuts	2018	■	■	■	■	■	■	■	J
85	4218	Cheesecake	2018	■	■	■					-
86	4220	Meringue cake	2018	■	■	■	■	■	■		A
87	4232	Coffe cake with not heat-treated cream	2018	■	■	■		■	■		D
88	4233	Polish chocolate cream cake	2018	■	■	■		■		■	E
89	4237	Cheesecake	2018	■	■	■		■	■		D
90	4238	Toffee cake	2018	■	■	■		■	■		D
91	4240	Toffee cake	2018	■	■	■		■			F
92	4241	Eclair	2018	■	■	■		■			F
93	4242	Cake	2018	■	■	■	■	■	■		A
94	4243	Cake	2018	■	■	■		■	■		D
95	4245	Cake	2018	■	■	■		■	■		D
96	4246	Napoleon Cake	2018	■	■	■	■	■	■		A
97	4266 A	Cake	2018	■	■	■	■	■			C
98	4266 B	Cake	2018	■	■	■		■			F
99	4267 A	Cake with cherry in alcohol	2018	■	■	■		■			F
100	4267 B	Cake with cherry in alcohol	2018	■	■	■					-
101	4268 A	Chocolate cake with cherry	2018	■	■	■		■	■		D
102	4292	Cake with cream	2018	■	■	■		■			F
103	4319 A	Sponge cake with cocoa and whipped cream	2019	■	■	■	■		■		I
104	4319 B	Sponge cake with cocoa and whipped cream	2019	■	■	■	■		■		I
105	4319 C	Sponge cake with cocoa and whipped cream	2019	■	■	■	■		■		I
106	4319 D	Sponge cake with cocoa and whipped cream	2019	■	■	■	■		■		I
107	4320 A	Polish chocolate cream cake	2019	■	■	■	■	■	■		A
108	4320 B	Polish chocolate cream cake	2019	■	■	■		■		■	E
109	4320 C	Polish chocolate cream cake	2019	■	■	■	■	■	■		A
110	4320 D	Polish chocolate cream cake	2019	■	■	■	■	■	■		A
111	4320 E	Polish chocolate cream cake	2019	■	■	■		■			F
112	4321	Cake with cream	2019	■	■	■	■	■	■		A

113	4322 A	Cake with cream	2019								A
114	4322 B	Cake with cream	2019								A
115	4322 C	Cake with cream	2019								A
116	4323 B	Cake	2019								A
117	4323 C	Cake	2019								A
118	4324 A	Sponge cake and poppy seed-coconut cake with jam and custard mass	2019								E
119	4324 B	Sponge cake and poppy seed-coconut cake with jam and custard mass	2019								A
120	4324 C	Sponge cake and poppy seed-coconut cake with jam and custard mass	2019								F
121	4324 D	Sponge cake and poppy seed-coconut cake with jam and custard mass	2019								F
122	4324 E	Sponge cake and poppy seed-coconut cake with jam and custard mass	2019								-
123	4325 C	Cake with cream	2019								F
124	4325 D	Cake with cream	2019								A
125	4325 E	Cake with cream	2019								F
126	4326 A	Napoleon Cake	2019								A
127	4326 B	Napoleon Cake	2019								A
128	4326 C	Napoleon Cake	2019								A
129	4326 D	Napoleon Cake	2019								A
130	4326 E	Napoleon Cake	2019								D
131	4327	Cooked pasta*	2019								D
132	4328 A	Chicken fricassee*	2019								E
133	4328	Chicken fricassee*	2019								A
134	4337	Salad with vegetables and meat*	2019								A
135	4339	Baked pâté*	2019								A
136	4348	Napoleon Cake	2019								C
137	4349	Eclair	2019								A
138	4371	Egg sandwich*	2019								I
139	4379	Wafer tubes with whipped cream	2019								D

140	4392	Cake	2019	■	■	■	■	■	■		A
141	4418	Cake with chocolate and whipped cream	2019	■	■	■		■	■		D
142	4420	Cake with chocolate and whipped cream	2019	■	■	■		■			F
143	4422	Cake with chocolate and whipped cream	2019	■	■	■	■	■	■		A
144	4423	Cake with chocolate and whipped cream	2019	■	■	■		■			F
145	4425	Blackberry pie	2019	■	■	■		■	■		D
146	4426	Blackberry pie	2019	■	■	■		■	■		D
147	4428	Blackberry pie	2019	■	■	■		■	■		D
148	4430	Tiramisu	2019	■	■	■		■			F
149	4431	Tiramisu	2019	■	■	■		■	■		D
150	4432	Choux pastry with custard cream	2019	■	■	■		■			F
151	4438	Brulee cheesecake	2019	■	■	■		■			F
152	4447	Chocolate cake with cream	2019	■	■	■	■	■	■		A
153	4448	Chocolate cake with cream	2019	■	■	■	■	■	■		A
154	4449	Chocolate cake with cream	2019	■	■	■	■	■	■		A
155	4450	Chocolate cake with cream	2019	■	■	■	■	■			C
156	4451	Chocolate cake with cream	2019	■	■	■	■	■	■		A
157	4470	Trout *	2019	■	■	■	■		■		I
158	4471	Tiramisu	2019	■	■	■		■			F
159	4473	Blackberry pie	2019	■	■	■		■			F
160	4476 A	Sponge cake and poppy seed-coconut cake with jam and cream	2019	■	■	■		■	■		D
161	4476 B	Sponge cake and poppy seed-coconut cake with jam and cream	2019	■	■	■		■	■		D
162	4476 D	Sponge cake and poppy seed-coconut cake with jam and cream	2019	■	■	■		■	■		D
163	4482	Tiramisu	2019	■	■	■	■	■			C
164	4483	Tiramisu	2019	■	■	■	■	■			C
165	4484	Tiramisu	2019	■	■	■	■	■			C
166	4486	Cake	2019	■	■	■	■	■	■		A
167	4487	Cake	2019	■	■	■	■	■	■		A

168	4488	Cake	2019								A
169	4489	Cake	2019								A
170	4499	Chocolate cake	2019								A
171	4500	Pizza*	2019								A
172	4501	Meringue and raspberry roulade	2019								A
173	4502	Cake	2019								A
174	4503	Coffee cake	2019								-
175	4508	Cake	2019								I
176	4509	Cake	2019								F
177	4510	Cake	2019								F
178	4512	Cake	2019								F
179	4513	Cake	2019								F
180	4514	Cake	2019								C
181	4515	Cake	2019								D
182	4522	Chocolate cake	2019								A
183	4524	Vegetable salad from frozen food*	2019								A
184	4525	Cocoa Cake	2019								A
185	4526	Cocoa Cake	2019								A
186	4530	Cake with nuts	2019								D
187	4531	Napoleon Cake	2019								A
188	4537 A	Polish chocolate cream cake	2019								E
189	4537 B	Polish chocolate cream cake	2019								B
190	4537 C	Polish chocolate cream cake	2019								E
191	4537 D	Polish chocolate cream cake	2019								E
192	4538	Passion fruit cake	2019								A
193	4539	Passion fruit cake	2019								A
194	4540	Passion fruit cake	2019								D
195	4542	Passion fruit cake	2019								F
196	4543	Caramel cake with peanuts	2019								C
197	4544	Caramel cake with peanuts	2019								C

198	4545	Caramel cake with peanuts	2019								-
199	4546	Caramel cake with peanuts	2019								D
200	4555	Toffi cake	2019								D
201	4557	Blackberry pie	2019								A
202	4558	Polish chocolate cream cake	2019								A
203	4566	Cake with cherry in alcohol	2019								D
204	4567	Cake with cherry in alcohol	2019								D
205	4568	Cake with cherry in alcohol	2019								G
206	4569	Cake with cherry in alcohol	2019								D
207	4570	Cake with cherry in alcohol	2019								A
208	4582	Cake with cherry in alcohol	2019								C
209	4590 A	Shortcrust pastry with honey, layered with semolina filling and covered with chocolate icing	2019								A
210	4590 B	Shortcrust pastry with honey, layered with semolina filling and covered with chocolate icing	2019								D
211	4590 C	Shortcrust pastry with honey, layered with semolina filling and covered with chocolate icing	2019								F
212	4590 D	Shortcrust pastry with honey, layered with semolina filling and covered with chocolate icing	2019								D
213	4590 E	Shortcrust pastry with honey, layered with semolina filling and covered with chocolate icing	2019								D
214	4594	Cake	2019								F
215	4604 A	Cake with whipped cream	2019								D
216	4604 B	Cake with whipped cream	2019								D
217	4604 C	Cake with whipped cream	2019								D
218	4604 D	Cake with whipped cream	2019								D
219	4604 E	Cake with whipped cream	2019								D
220	4605 A	Napoleon Cake	2019								D

221	4605 B	Napoleon Cake	2019								F
222	4605 C	Napoleon Cake	2019								F
223	4606 A	Cake with whipped cream	2019								F
224	4606 B	Cake with whipped cream	2019								G
225	4606 C	Cake with whipped cream	2019								A
226	4606 D	Cake with whipped cream	2019								C
227	4607 A	Cake with whipped cream	2019								F
228	4607 B	Cake with whipped cream	2019								E
229	4607 C	Cake with whipped cream	2019								A
230	4607 D	Cake with whipped cream	2019								F
231	4608 A	Napoleon Cake	2019								F
232	4608 B	Napoleon Cake	2019								A
233	4608 C	Napoleon Cake	2019								A
234	4608 D	Napoleon Cake	2019								C
235	4608 E	Napoleon Cake	2019								F
236	4611 A	Napoleon Cake	2019								A
237	4612A	Cake with whipped cream and fruit	2019								D
238	4612 B	Cake with whipped cream and fruit	2019								F
239	4612 C	Cake with whipped cream and fruit	2019								F
240	4612 D	Cake with whipped cream and fruit	2019								F
241	4614 A	Cake with whipped cream	2019								D
242	4614 B	Cake with whipped cream	2019								D
243	4614 E	Cake with whipped cream	2019								A
244	4615 A	Cake with pudding	2019								A
245	4616 A	Meter-long cake	2019								D
246	4616 B	Meter-long cake	2019								A
247	4618 A	Cupcake cake with pudding and fruit	2019								I
248	4618 B	Cupcake cake with pudding and fruit	2019								F
249	4618 C	Cupcake cake with pudding and fruit	2019								A
250	4618 D	Cupcake cake with pudding and fruit	2019								A

251	4618 E	Cupcake cake with pudding and fruit	2019	■	■	■	■	■	■		A
252	4623 A	Polish chocolate cream cake	2019	■	■	■		■			F
253	4625 A	Eclair	2019	■	■	■	■	■	■		A
254	4625 B	Eclair		■	■	■	■	■	■		A
255	4625 C	Eclair	2019	■	■	■		■			F
256	4626	Cupcake with pudding cream	2019	■	■	■	■	■	■		A
257	4642	Chocolate cake with cream	2019	■	■	■		■	■		D
258	4643	Chocolate cake with cream	2019	■	■	■		■	■		D
259	4644	Cake with not heat-treated cream	2019	■	■	■		■		■	E
260	4645	Cake with not heat-treated cream	2019	■	■	■		■			F
261	4646	Cake with not heat-treated cream	2019	■	■	■		■			F
262	4647	Cake with not heat-treated cream	2019	■	■	■		■			F
263	4648	Roulade cake with whipped cream	2019	■	■	■	■	■			C
264	4657 A	Cake with whipped cream	2019	■	■	■	■	■	■		A
265	4657 B	Cake with whipped cream	2019	■	■	■		■			F
266	4657 C	Cake with whipped cream	2019	■	■	■		■			F
267	4657 D	Cake with whipped cream	2019	■	■	■		■			F
268	4657 E	Cake with whipped cream	2019	■	■	■		■			F
269	4668	Chocolate cake with cherry	2019	■	■	■		■			F
270	4679 A	Cake with whipped cream	2019	■	■	■	■	■			C
271	4679 B	Cake with whipped cream	2019	■	■	■	■	■			C
272	4679 C	Cake with whipped cream	2019	■	■	■	■	■			C
273	4679 D	Cake with whipped cream	2019	■	■	■	■	■			C
274	4679 E	Cake with whipped cream	2019	■	■	■	■	■			C
275	4686 A	Cupcake cake with pudding	2019	■	■	■		■			F
276	4686 B	Cupcake cake with pudding	2019	■	■	■	■	■	■		A
277	4686 C	Cupcake cake with pudding	2019	■	■	■		■		■	E
278	4686 E	Cupcake cake with pudding	2019	■	■	■		■		■	E
279	4687	Polish chocolate cream cake	2019	■	■	■	■	■	■		A
280	4688	Sponge cake with custard cream	2019	■	■	■	■	■	■		A

281	4699 A	Cake with cream	2019								F
282	4699 B	Cake with cream	2019								A
283	4699 C	Cake with cream	2019								F
284	4699 D	Cake with cream	2019								F
285	4699 E	Cake with cream	2019								F
286	4706 A	Meter-long cake	2019								H
287	4706 B	Meter-long cake	2019								F
288	4706 D	Meter-long cake	2019								F
289	4707 A	Coconut cake	2019								F
290	4707 B	Coconut cake	2019								F
291	4707 C	Coconut cake	2019								F
292	4707 D	Coconut cake	2019								F
293	4707 E	Coconut cake	2019								F
294	4708 A	Mrs Walewska cake	2019								F
295	4708 B	Mrs Walewska cake	2019								A
296	4708 C	Mrs Walewska cake	2019								D
297	4708 D	Mrs Walewska cake	2019								A
298	4708 E	Mrs Walewska cake	2019								F
299	4725	Meringues with cocoa cream	2019								C
300	4735	Cake with whipped cream	2019								F
301	4737	Strawberry roulade with mascarpone	2019								C
302	4738	Marshmallow in chocolate	2019								C
303	4739	Blueberry pie	2019								C
304	4741	Cheesecake with peaches	2019								A
305	4742	Sponge cake with whipped cream	2019								-
306	4743	Blackberry pie	2019								F
307	4744	Cake	2019								C
308	4746	Cake	2019								C
309	4747	Coconut cake	2019								C
310	4748	Coconut cake	2019								C

311	4749	Napoleon Cake	2019	■	■	■	■	■	■		A
312	4750	Cake with whipped cream	2019	■	■	■		■			F
313	4752	Eclair with whipped cream	2019	■	■	■			■		G
314	4753	Yogurt cake	2019	■	■	■	■	■	■		A
315	4754	Cream cake	2019	■	■	■	■	■	■		A
316	4759	Tiramisu	2019	■	■	■		■		■	E
317	4760	Tiramisu	2019	■	■	■		■		■	E
318	4761	Cake	2019	■	■	■	■	■	■		A
319	4763	Meringue cake	2019	■	■	■		■			F
320	4765	Cocoa cheesecake	2019	■	■	■					-
321	4766	Eclair	2019	■	■	■		■		■	E
322	4767	Dried fruit cake	2019	■	■	■					-
323	4771	Cheesecake	2019	■	■	■	■	■			C
324	4772	Eclair	2019	■	■	■	■	■	■		A
325	4773	Polish chocolate cream cake	2019	■	■	■	■	■	■		A
326	4774	Eclair	2019	■	■	■		■			F
327	4775	Eclair	2019	■	■	■		■			F
328	4776	Napoleon Cake	2019	■	■	■	■	■	■		A
329	4778	Pear cake	2019	■	■	■	■	■	■		A
330	4779	Rolls with pudding	2019	■	■	■	■	■	■		A
331	4806 A	Toffee cake	2019	■	■	■		■	■		D
332	4806 B	Toffee cake	2019	■	■	■		■	■		D
333	4806 C	Toffee cake	2019	■	■	■		■	■		D
334	4806 D	Toffee cake	2019	■	■	■		■	■		D
335	4806 E	Toffee cake	2019	■	■	■		■			F
336	4807	Meatballs*	2019	■	■	■	■	■	■		A
337	4808	Bulgur groats*	2019	■	■	■	■	■			C
338	4809 A	Eclair	2019	■	■	■		■	■		D
339	4809 B	Eclair	2019	■	■	■		■			F
340	4809 C	Eclair	2019	■	■	■		■			F

341	4809 D	Eclair	2019								C
342	4809 E	Eclair	2019								D
343	4810	Mrs Walewska cake	2019								C
344	4811	Tiramisu	2019								F
345	4812	Vegetable stew with chicken *	2019								F
346	4813	Green vegetable soup *	2019								F
347	4814 A	Fish chop *	2019								A
348	4816 A	Cake with cream	2019								C
349	4816 C	Cake with cream	2019								A
350	4816 D	Cake with cream	2019								E
351	4816 E	Cake with cream	2019								A
352	4817 A	Sponge cake and poppy seed-coconut cake with jam and custard mass	2019								C
353	4817 B	Sponge cake and poppy seed-coconut cake with jam and custard mass	2019								C
354	4817 C	Sponge cake and poppy seed-coconut cake with jam and custard mass	2019								A
355	4817 D	Sponge cake and poppy seed-coconut cake with jam and custard mass	2019								C
356	4817 E	Sponge cake and poppy seed-coconut cake with jam and custard mass	2019								F
357	4818 A	Chocolate cake	2019								F
358	4818 B	Chocolate cake	2019								F
359	4818 C	Chocolate cake	2019								A
360	4818 D	Chocolate cake	2019								C
361	4818 E	Chocolate cake	2019								A
362	4819 A	Cake	2019								A
363	4819 B	Cake	2019								A
364	4819 C	Cake	2019								A
365	4819 D	Cake	2019								A
366	4820	Spinach *	2019								F
367	4839	Toffee cake	2019								A

368	4840	Toffee cake	2019								C
369	4846	Coconut cake	2019								A
370	4847	Coconut cake	2019								C
371	4848	Coconut cake	2019								A
372	4848	Coconut cake	2019								F
373	4849	Coconut cake	2019								C
374	4850	Coconut cake	2019								C
375	4851	White cabbage salad*	2019								A
376	4852	Garlic sauce*	2019								I
377	4854	Pasta with spinach and chicken*	2019								C
378	4855	Coconut cake	2019								F
379	4856	Radish sprouts*	2019								C
380	4857	Chives*	2019								C
381	4858	Chives*	2019								C
382	4861 B	Cake	2019								E
383	4863	Chives*	2019								-
384	4865 A	Cookies	2019								A
385	4865 B	Cookies	2019								A
386	4865 C	Cookies	2019								A
387	4865 D	Cookies	2019								A
388	4865 E	Cookies	2019								A
389	4867	Strawberry cake	2019								F
390	4868	Strawberry cake	2019								F
391	4869	Strawberry cake	2019								F
392	4870	Strawberry cake	2019								F
393	4871	Cakes	2019								F
394	4872	Cakes	2019								D
395	4873	Cakes	2019								F
396	4874	Cakes	2019								F
397	4875	Cheesecake with peaches	2019								F

398	4876	Toffee cake	2019	■	■	■		■			F
399	4877	Toffee cake	2019	■	■	■		■			F
400	4878	Toffee cake	2019	■	■	■		■			F
401	4879	Toffee cake	2019	■	■	■		■			F
402	4880	Raspberry cake	2019	■	■	■		■			F
403	4881	Raspberry cake	2019	■	■	■		■	■		D
404	4882	Raspberry cake	2019	■	■	■		■	■		D
405	4883	Raspberry cake	2019	■	■	■	■	■			C
406	4887	Cheesecake	2019	■	■	■	■	■	■		A
407	4889	Biscuit roulade	2019	■	■	■	■	■			C
408	4890	Biscuit roulade	2019	■	■	■	■	■			C
409	4891	Biscuit roulade	2019	■	■	■	■	■	■		A
410	4892	Biscuit roulade	2019	■	■	■	■	■			C
411	4893	Biscuit roulade	2019	■	■	■	■	■			C
412	4908 A	Mrs Walewska cake	2019	■	■	■		■			F
413	4908 B	Mrs Walewska cake	2019	■	■	■		■			F
414	4908 C	Mrs Walewska cake	2019	■	■	■		■			F
415	4908 D	Mrs Walewska cake	2019	■	■	■		■			F
416	4911	Sponge cake roll with cream	2019	■	■	■	■	■	■		A
417	4912 A	Chocolate cookie cake with cream	2019	■	■	■	■	■	■		A
418	4912 B	Chocolate cookie cake with cream	2019	■	■	■		■			F
419	4912 C	Chocolate cookie cake with cream	2019	■	■	■		■		■	E
420	4912 D	Chocolate cookie cake with cream	2019	■	■	■		■			F
421	4912 E	Chocolate cookie cake with cream	2019	■	■	■	■	■			C
422	4913 A	Currant pie	2019	■	■	■	■	■	■		A
423	4913 B	Currant pie	2019	■	■	■		■			F
424	4913 C	Currant pie	2019	■	■	■		■			F
425	4916	Cake	2019	■	■	■					-
426	4917 A	Plum cake in alcohol	2019	■	■	■	■	■	■		A
427	4917 B	Plum cake in alcohol	2019	■	■	■		■			F

428	4922 A	Cake	2019								-
429	4922 B	Cake	2019								A
430	4922 C	Cake	2019								-
431	4924	Cake with whipped cream	2019								A
432	4925	Tiramisu	2019								A
433	4978	Sponge cake roll with jelly	2019								D
434	4984	Cream puff	2019								D
435	4985	Eclairs with cream	2019								F
436	4986	Strawberry cake	2019								F
437	4987	Cake with nuts	2019								F
438	4988	Polish chocolate cream cake	2019								A
439	4989	Toffee cake	2019								A
440	4990	Cocoa sponge cake	2019								A
441	4991	Toffee cupcake	2019								C
442	4993	Cake with whipped cream and chocolate	2019								A
443	5002 A	Honey cake tops filled with custard and kajmak cream, nuts and chocolate icing.	2019								-
444	5002 B	Honey cake tops filled with custard and kajmak cream, nuts and chocolate icing.	2019								A
445	5002 C	Honey cake tops filled with custard and kajmak cream, nuts and chocolate icing.	2019								-
446	5002 D	Honey cake tops filled with custard and kajmak cream, nuts and chocolate icing.	2019								F
447	5002 E	Honey cake tops filled with custard and kajmak cream, nuts and chocolate icing.	2019								D
448	5003	Horseradish sauce *	2019								C
449	5010	Cake with whipped cream	2019								D
450	5014	Cake with whipped cream	2019								D
451	5015	Caramel cake with peanuts	2019								-
452	5033 A	Cake with raspberries, whipped cream and meringue	2020								C
453	5034 A	Cake with raspberries, whipped cream and meringue	2020								D

454	5034 B	Coconut cake	2020								D
455	5034 C	Coconut cake	2020								C
456	5034 E	Coconut cake	2020								C
457	5050	Yeast cake with custard cream	2020								A
458	5051 A	Cake with jelly and strawberry	2020								D
459	5051 B	Cake with jelly and strawberry	2020								A
460	5054	Cake with not heat-treated cream	2020								C
461	5060	Milk soup*	2020								-
462	5061	Apple pie cake	2020								A
463	5062	Cake	2020								C
464	5063	Cake	2020								G
465	5064	Cake	2020								A
466	5065	Elderberry cake	2020								C
467	5066	Cake with whipped cream and chocolate	2020								D
468	5074 A	Almond cake	2020								F
469	5074 C	Almond cake	2020								F
470	5074 D	Almond cake	2020								F
471	5074 E	Almond cake	2020								F
472	5080 A	Napoleon Cake	2020								F
473	5080 B	Napoleon Cake	2020								D
474	5081	Toffee cake	2020								-
475	5082 A	Biscuit roulade	2020								A
476	5082 B	Biscuit roulade	2020								G
477	5082 C	Biscuit roulade	2020								G
478	5082 D	Biscuit roulade	2020								A
479	5082 E	Eclair	2020								D
480	5083 C	Eclair	2020								-
481	5083 D	Eclair	2020								-
482	5084 A	Cake with whipped cream	2020								I
483	5084 B	Cake with whipped cream	2020								A

484	5084 C	Cake with whipped cream	2020	■	■	■		■			F
485	5085 A	Biscuit roulade	2020	■	■	■	■		■		I
486	5085 B	Biscuit roulade	2020	■	■	■		■			F
487	5085 C	Biscuit roulade	2020	■	■	■	■	■			C
488	5085 D	Biscuit roulade	2020	■	■	■	■	■			C
489	5086 A	Eclair	2020	■	■	■		■	■		D
490	5086 B	Eclair	2020	■	■	■					-
491	5086 C	Eclair	2020	■	■	■					-
492	5088 A	Fruit cake	2020	■	■	■		■			F
493	5088 B	Fruit cake	2020	■	■	■	■	■			C
494	5088 C	Fruit cake	2020	■	■	■	■	■	■		A
495	5088 D	Fruit cake	2020	■	■	■		■	■		D
496	5088 E	Fruit cake	2020	■	■	■	■				H
497	5090 A	Coconut cake	2020	■	■	■	■	■			C
498	5090 B	Coconut cake	2020	■	■	■					-
499	5090 C	Coconut cake	2020	■	■	■		■			F
500	5090 D	Coconut cake	2020	■	■	■	■	■	■		A
501	5090 E	Coconut cake	2020	■	■	■	■	■			C
502	6007 A	Cake with cream	2020	■	■	■		■	■		D
503	6007 B	Cake with cream	2020	■	■	■		■	■		D
504	6007 C	Cake with cream	2020	■	■	■		■	■		D
505	6007 E	Cake with cream	2020	■	■	■		■	■		D
506	6008 A	Cake with cream	2020	■	■	■		■	■		D
507	6008 B	Cake with cream	2020	■	■	■		■	■		D
508	6008 C	Cake with cream	2020	■	■	■		■	■		D
509	6008 D	Cake with cream	2020	■	■	■		■	■		D
510	6008 E	Cake with cream	2020	■	■	■	■	■	■		A
511	6019 A	Toffee macarons	2020	■	■	■	■	■			C
512	6019 B	Toffee macarons	2020	■	■	■	■	■			C
513	6019 C	Toffee macarons	2020	■	■	■	■	■			C

514	6019 D	Toffee macarons	2020								A
515	6029	Bun with pudding	2020								F
516	6037	Wafer tubes with marzipan and raspberry	2020								F
517	6040	Wafer tubes with marzipan and raspberry	2020								C
518	6041	Wafer tubes with marzipan and raspberry	2020								C
519	6063 A	Cake with not heat-treated cream	2020								F
520	6063 B	Cake with not heat-treated cream	2020								F
521	6063 C	Cake with not heat-treated cream	2020								F
522	6063 D	Cake with not heat-treated cream	2020								F
523	6063 E	Cake with not heat-treated cream	2020								F
524	6065 A	Cake with not heat-treated cream	2020								F
525	6065 B	Cake with not heat-treated cream	2020								F
526	6065 C	Cake with not heat-treated cream	2020								D
527	6065 D	Cake with not heat-treated cream	2020								F
528	6065 E	Cake with not heat-treated cream	2020								F
529	6066 A	Cake with not heat-treated cream	2020								A
530	6066 B	Cake with not heat-treated cream	2020								F
531	6066 C	Cake with not heat-treated cream	2020								F
532	6066 D	Cake with not heat-treated cream	2020								C
533	6066 E	Cake with not heat-treated cream	2020								A
534	6069	Cake	2020								-
535	6070	Fruit cake	2020								F
536	6071	Currant pie	2020								C
537	6072	Cake	2020								C
538	6073	Cake with whipped cream and chocolate	2020								F
539	6074	Bun with pudding	2020								C
540	6075	Fruit cake	2020								C
541	6115 B	Mandarin dessert	2020								D
542	6116	Mascarpone cake with strawberries	2020								C
543	6117	Cheesecake	2020								F

544	6118	Marshmallow in chocolate	2020	■	■	■		■			F
545	6119	Shortcrust pastry with cheese	2020	■	■	■	■	■	■		A
546	6120	Yeast Rolls	2020	■	■	■	■	■			C
547	6121	Cake with raspberries, whipped cream and meringue	2020	■	■	■	■	■			C
548	6123	Coconut cake	2020	■	■	■		■	■		D
549	6115 A	Mandarin dessert	2020	■	■	■		■			F
550	6122	Napoleon Cake	2020	■	■	■		■			F

black boxes – gene presence, white boxes- gene absence

Supplementary Table S2. Taxonomic classification of *isolates* from RTE food (n=50)

	Strain	Region of isolation	Source	Year of isolation	Phylogenic group (Clades *panC* group)	Species (ANI) Btyper (%)	Subspecies (ANI) Btyper (%)	Final taxon names Btyper	Species Ridom (%)
1	3988 A	Masovia	Cake with non-heat-treated cream	2018	IV	*B. cereus s.s.* (98)	No subspecies	*B. cereus s.s.*	*B. cereus* (99)
2	3992 B	Masovia	Cake with non-heat-treated cream	2018	III	*B. mosaicus* (94)	No subspecies	*B. mosaicus*	*B. cereus* (99)
3	3996 A	Masovia	Cake with non-heat-treated cream	2018	III	*B. mosaicus* (98)	cereus (100)	*B. mosaicus* subsp. cereus biovar Emeticus; *B. cereus* biovar Emeticus; *B.* Emeticus	*B. cereus* (100)

4	4011	Masovia	Alpine cake	2018	IV	*B. cereus s.s.* (99)	No subspecies	*B. cereus s.s.* biovar Thuringiensis; *B.* Thuringiensis	*B. thuringiensis* (100)
5	4022	Łódź	Coconut cake	2018	IV	*B. cereus s.s.* (99)	No subspecies	*B. cereus s.s.*	*B. cereus* (100)
6	4031	Łódź	Cake with cream and apples	2018	III	*B. mosaicus* (98)	No subspecies	*B. mosaicus*	*B. thuringiensis* (99)
7	4032	Lublin	Mrs. Walewska cake	2018	IV	*B. cereus s.s.* (99)	No subspecies	*B. cereus s.s.*	*B. bombysepticus (99)*
8	4036	Lublin	Cake with not heat-treated cream	2018	IV	*B. cereus s.s.* (97)	No subspecies	*B. cereus s.s.*	*B. thuringiensis* (99)
9	4051	Masovia	Strawberry cake	2018	II	*B. mosaicus* (95)	No subspecies	*B. mosaicus*	*B. cereus* (97)
10	4109	Greater Poland	Cake with cream	2018	II	*B. mosaicus* (93)	No subspecies	*B. mosaicus*	*B. wiedmannii* (97)
11	4136	Opole	Sponge-fat cake with red currant gel and toffee-flavoured	2018	III	*B. mosaicus* (94)	No subspecies	*B. mosaicus* biovar Emeticus; *B.* Emeticus	*B. cereus* (99)
12	4144	Opole	Peach cake	2018	IV	*B. cereus s.s.* (98)	No subspecies	*B. cereus s.s.*	*B. cereus* (99)

13	4201	Lower Silesia	Cream roulade with non-heat-treated cream	2018	II	*B. mosaicus* (93)	No subspecies	*B. mosaicus*	*B. wiedmannii* (99)
14	4204	Lower Silesia	Cream roulade with non-heat-treated cream	2018	IV	*B. cereus s.s.* (98)	cereus (99)	*B. mosaicus* subsp. cereus biovar Emeticus; *B. cereus* biovar Emeticus; *B.* Emeticus	*B. cereus* (100)
15	4217	Lower Silesia	Cake with nuts	2018	IV	*B. mosaicus* (94)	cereus (98)	*B. mosaicus* subsp. cereus biovar Emeticus; *B. cereus* biovar Emeticus; *B.* Emeticus	*B. cereus* (100)
16	4220	Lower Silesia	Meringue cake	2018	III	*B. cereus s.s.* (97)	No subspecies	*B. cereus s.s.* biovar Emeticus*; *B.* Emeticus*	*B. cereus* (100)
17	4320 B	Masovia	Polish chocolate cream cake	2019	III	*B. mosaicus* (95)	cereus (100)	*B. mosaicu*s subsp. cereus biovar Emeticus; *B. cereus* biovar Emeticus; *B.* Emeticus	*B. cereus* (100)
18	4327	Masovia	Cooked pasta	2019	III	*B. mosaicus* (95)	No subspecies	*B. mosaicus*	*B. paranthracis* (98)

19	4348	Lower Silesia	Cakes with heat treatment cream - Napoleon Cake	2019	V	*B. toyonensis* (99)	No subspecies	*B. toyonensis* biovar Thuringiensis; *B.* Thuringiensis	*B. toyonensis* (99)
20	4422	Masovian	Cake with chocolate and whipped cream	2019	IV	*B. cereus s.s.* (97)	No subspecies	*B. cereus s.s.*	*B. cereus* (100)
21	4537 A	Łódź	Polish chocolate cream cake	2019	III	*B. mosaicus* (95)	cereus (100)	*B. mosaicus* subsp. cereus biovar Emeticus; *B. cereus* biovar Emeticus; *B.* Emeticus	*B. paranthracis* (100)
22	4607 B	West Pomeranian	Cake with whipped cream	2019	III	*B. mosaicus* (95)	cereus (100)	*B. mosaicus* subsp. cereus biovar Emeticus; *B. cereus* biovar Emeticus; *B.* Emeticus	*B. cereus* (100)
23	4618 B	Łódź	Cupcake cake with pudding and fruit	2019	III	*B. mosaicus* (94)	No subspecies	*B. mosaicus*	*B. cereus* (100)
24	4644	Łódź	Cake with not heat-treated cream	2019	III	*B. mosaicus* (95)	cereus (100)	*B. mosaicus* subsp. cereus biovar Emeticus; *B. cereus* biovar	*B. paranthracis* (100)

								Emeticus; *B.* Emeticus	
25	4686 B	Łódź	Cupcake cake with pudding	2019	IV	*B. cereus s.s.* (97)	No subspecies	*B. cereus s.s.*	*B. cereus* (100)
26	4753	Lower Silesia	Yogurt cake	2019	IV	*B. cereus s.s.* (96)	No subspecies	*B. cereus s.s.* biovar Thuringiensis; *B.* Thuringiensis	*B. thuringiensis* (100)
27	4763	Lower Silesia	Meringue cake	2019	III	*B. mosaicus* (98)	No subspecies	*B. mosaicus*	*B. thuringiensis* (99)
28	4810	Masovia	Mrs. Walewska cake	2019	II	*B. mosaicus* (93)	No subspecies	*B.* mosaicus	*B. wiedmannii* (99)
29	4818 C	Masovia	Chocolate cake	2019	IV	*B. cereus s.s.* (97)	No subspecies	*B. cereus s.s.*	*B. cereus* (100)
30	4819 A	Masovia	Cake	2019	IV	*B. cereus s.s.* (97)	No subspecies	*B. cereus s.s.*	*B. cereus* (100)
31	4865 C	Greater Poland	Cookies	2019	IV	*B. cereus s.s.* (98)	No subspecies	*B. cereus s.s.*	*B. thuringiensis* (99)
32	4912 A	Łódź	Chocolate cookie cake with cream	2019	IV	*B. cereus s.s.* (99)	No subspecies	*B. cereus s.s.*	*B. bombysepticus* (99)
33	4913 A	Łódź	Currant pie	2019	IV	*B. cereus s.s.* (99)	No subspecies	*B. cereus s.s.*	*B. bombysepticus* (99)
34	5034 A	Masovian	Cake with raspberries, whipped	2019	III	*B. mosaicus* (98)	No subspecies	*B. mosaicus*	*B. anthracis* (98)

			cream and meringue						
35	5051 B	Lubelskie	Cake with jelly and strawberry	2019	V	*B. toyonensis* (98)	No subspecies	*B. toyonensis*	*B. toyonensis* (99)
36	5062	Greater Poland	Cake	2020	V	*B. mycoides* (94)	No subspecies	*B. mycoides*	*B. mycoides* (99)
37	5065	Greater Poland	Elderberry cake	2020	VI	*B. cereus s.s.* (97)	No subspecies	*B. cereus s.s.*	*B. cereus* (100)
38	5080 B	Łódź	Napoleon cake	2020	IV	*B. mosaicus* (94)	No subspecies	*B. mosaicus*	*B. cereus* (99)
39	5082 A	Łódź	Biscuit roulade	2020	IV	*B. cereus s.s.* (98)	No subspecies	*B. cereus s.s.*	*B. bombysepticus* (99)
40	5084 B	Łódź	Cake with whipped cream	2020	IV	*B. cereus s.s.* (97)	No subspecies	*B. cereus s.s.*	*B. cereus* (100)
41	5085 D	Łódź	Biscuit roulade	2020	IV	*B. cereus s.s.* (98)	No subspecies	*B. cereus s.s.*	*B. cereus* (99)
42	5086 B	Łódź	Eclair	2020	Unknown	Species unknown	No subspecies	(Species unknown)	*B. pumilus* (98)
43	5088 A	Łódź	Fruit cake	2019	III	*B. mosaicus* (95)	cereus (97)	*B. mosaicus* subsp. cereus; *B. cereus*	*B. paranthracis* (99)
44	5090 C	Świętokrzyskie	Coconut cake	2019	III	*B. mosaicus* (95)	cereus (99)	*B. mosaicus* subsp. cereus; *B. cereus*	*B. cereus* (99)
45	6008 A	Varmian-Masurian	Cake with cream	2020	IV	*B. cereus s.s.* (98)	No subspecies	*B. cereus s.s.*	*B. cereus* (99)

46	6019 A	Podkarpackie	Toffee macarons	2020	II	*B. mosaicus* (93)	No subspecies	*B. mosaicus*	*B. wiedmannii* (97)
47	6029	Lublin	Bun with pudding	2020	II	*B. mosaicus* (93)	No subspecies	*B. mosaicus*	*B. mobilis* (97)
48	6066 A	Greater Poland	Cake with not heat-treated cream	2020	IV	*B. cereus s.s.* (97)	No subspecies	*B. cereus s.s.*	*B. cereus* (100)
49	6071	Greater Poland	Currant cake	2020	II	*B. mosaicus* (93)	No subspecies	*B. mosaicus*	*B. wiedmannii* (97)
50	6073	Greater Poland	Cake with whipped cream and chocolate	2020	II	*B. mosaicus* (93)	No subspecies	*B. mosaicus*	*B. mobilis* (97)
